# Supplementary figures and images for: Identification of Estrogen Signaling in a Prioritization Study of Intraocular Pressure-Associated Genes
Source: Int J Mol Sci. 2021 Sep 24;22(19):10288. doi: 10.3390/ijms221910288 (PMC8508848; doi:10.3390/ijms221910288)

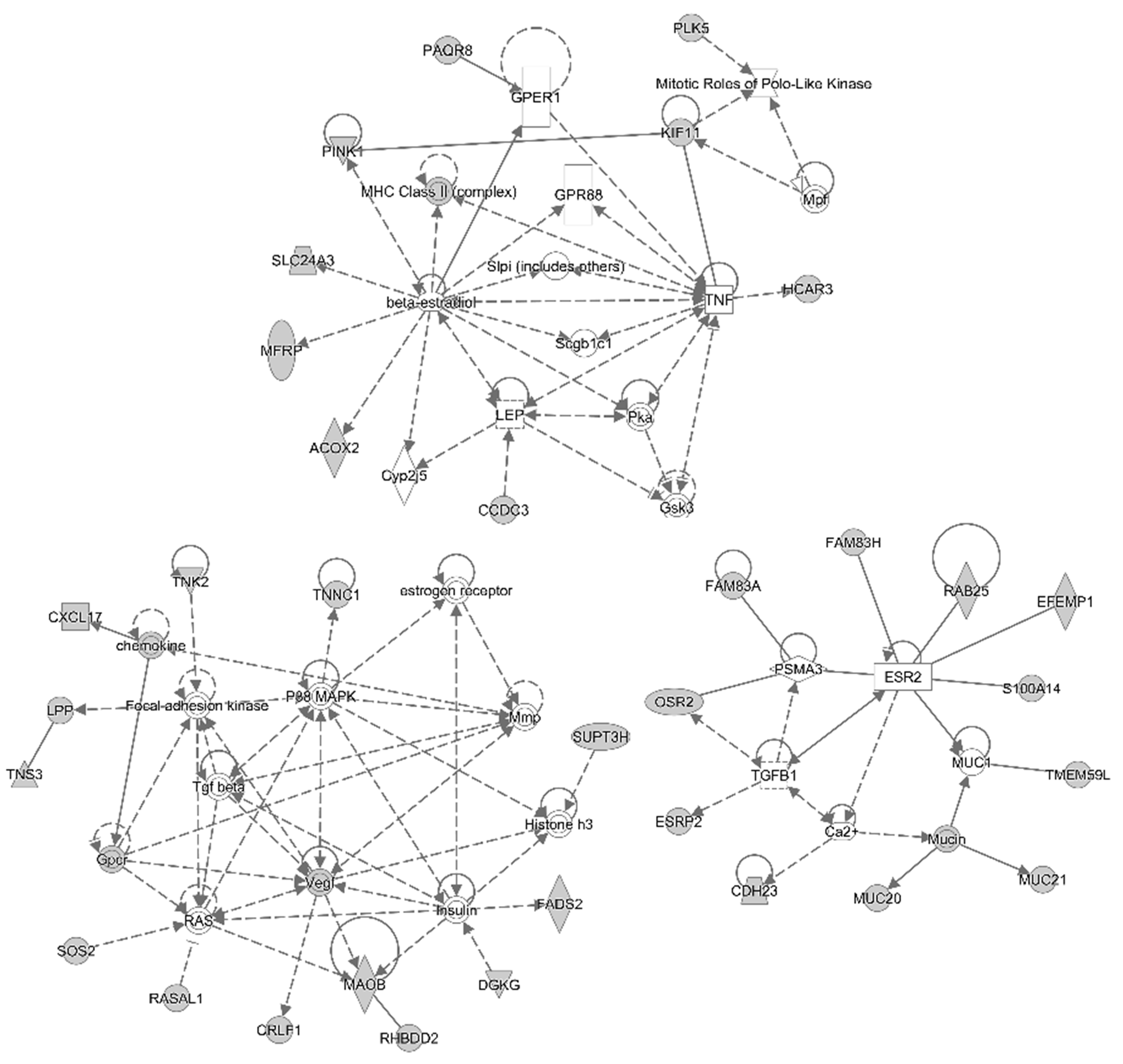

Supplement: Supplementary file 1 [file ijms-22-10288-s001.zip › SupplementalFigureS1.tif]
